# Destination Airway: Tracking Granulocytes in Asthma

**DOI:** 10.1016/j.ebiom.2014.11.002

**Published:** 2014-11-07

**Authors:** Christopher E. Brightling

**Affiliations:** Department of Infection, Inflammation and Immunity, Institute for Lung Health, University of Leicester, Leicester LE3 9QP, UK

Asthma affects over 300 million people worldwide and its prevalence continues to rise. It is typically characterised by intermittent symptoms of breathlessness, cough and wheeze punctuated with asthma attacks together with variable airflow obstruction, which are more frequent and persistent in severe disease (Chung et al., 2014). Underpinning this clinical presentation is airway inflammation and remodelling (Hartley et al., 2014).

Airway inflammation in asthma is typically eosinophilic in association with allergic sensitisation, particularly in those with early onset disease (Chung et al., 2014, Hartley et al., 2014). Allergen challenge is an established asthma model with features of both an early, predominately mast cell mediated, and a late asthmatic response driven by a more complex inflammatory response characterised by eosinophilic inflammation. However, beyond allergic asthma there is an increasing recognition that the inflammatory response in asthma is more heterogeneous with both eosinophilic and neutrophilic inflammation mediated by a combination of Th1/Tc1, Th2 and Th17 cytokines. Importantly, these inflammatory profiles do not necessarily occur independently, but may co-exist to varying degrees within an individual over time. The aetiology of neutrophilic asthma is poorly understood and is likely in part due to effects of high dose corticosteroid therapy, exposure to pollutants and pathogens (Hartley et al., 2014). Although the cause of eosinophilic and neutrophilic inflammatory responses in an individual are not fully understood granulocyte trafficking has been studied extensively and the critical growth factors, cytokines, chemokines and adhesion molecules have been described that coordinate hematopoiesis, bone marrow egression, adhesion to the endothelium, selective chemotaxis and survival in tissue (Hartley et al., 2014).

Our understanding of granulocyte trafficking has underpinned the development of several biologic and small molecule therapies targeting granulocyte recruitment and survival in asthma including anti-IL5, anti-CRTh2, anti-CCR3, and anti-CXCR2 (Chung et al., 2014, Hartley et al., 2014, Nair et al., 2012, Haldar et al., 2009, Ortega et al., 2014). It is therefore perhaps somewhat surprising that our knowledge of the dynamics of granulocyte trafficking in humans in both health and disease remains limited and that this lack of understanding not only impacts upon a more comprehensive insight into mechanisms of granulocyte migration, but also challenges our understanding of the relative importance of different potential therapeutic targets. With several new therapies in late phase development this greater insight would help to better position these therapies and inform the development of others. Therefore safe and reliable systems to track granulocytes in asthma are required.

To address this challenge Lukawska and colleagues report in this issue of EBioMedicine a small proof-of-concept study of real time, differential tracking of human eosinophil and neutrophil migration in 12 atopic asthmatics following allergen challenge (Lukawska et al., 2014). This is an extension of their earlier work reported with healthy volunteers (Lukawska et al., 2014) and is similar to reports by others exploring granulocyte trafficking in health (Farahi et al., 2012, Farahi et al., 2014). They purified eosinophil and neutrophils from the asthmatic subjects radiolabelled them, re-infused the cells and tracked the granulocyte kinetics by scintigraphy. They found that neutrophil efflux in the lungs was slower than eosinophils and systemic corticosteroid reduced lung retention of eosinophils albeit non-significantly. The study was small and underpowered to observe important clinical changes following allergen challenge to associate with the granulocyte trafficking, but does provide confidence for further clinical development. The study focused upon allergen challenge, but to understand granulocyte dynamics other established models would be valuable to explore such as viral or lipopolysaccharide challenges. The approach described by Lukawska and colleagues also increased activation of eosinophils and neutrophils which limits exploration of trafficking in health whereby the cells are unlikely to be activated (Farahi et al., 2012), but whether this is a major limitation in disease needs to be further explored.

*In vivo* real time tracking of granulocytes has immediate impact upon early phase clinical development as it can be incorporated into model systems to explore the effects of therapies upon granulocyte migration. However, with an increasing number of potential targets understanding their relative importance in different model systems and ultimately in specific patient subgroups is critical and is the drive towards stratified medicine. One approach to begin to dissect this complexity is multi-scale computational modelling, which might provide some important insights and become key in drug development and clinical decisions (Burrowes et al., 2013). These multi-scale models can be applied to predict relative mechanistic responses to therapies *in silico*. Intervention studies can then inform, validate and further extend these models to improve their clinical utility. This parallel approach of *in silico* modelling with *in vivo* experimental approaches that closely reflect the disease will inform asthma mechanisms and improve a stratified approach to therapy. Therefore, understanding the dynamics of the traffic control system in asthma to direct granulocytes to their airway destination continues to be an important focus of research.

## Conflicts of Interest

CEB has received grants and consultancy from AstraZeneca/MedImmune, GlaxoSmithKline, Novartis, Roche/Genentech, Almirall, Chiesi, and Boehringer-Ingelheim.
